# Full**-**Genome Sequence of Hibiscus Chlorotic Ringspot Virus from Israel

**DOI:** 10.1128/genomeA.01050-13

**Published:** 2013-12-12

**Authors:** Neta Luria, Victoria Reingold, Oded Lachman, Aviv Dombrovsky

**Affiliations:** Department of Plant Pathology, ARO, the Volcani Center, Bet Dagan, Israel

## Abstract

*Hibiscus rosa-sinensis* is one of the most prevalent ornamental plants grown in private and public gardens. Hibiscus chlorotic ringspot virus (HCRSV) is a member of the *Carmovirus* genus, with a positive single-strand RNA that putatively encodes seven proteins. The complete genome of the first Israeli isolate of HCRSV, HCRSV-IL, comprises 3,908 nucleotides and shows 93% nucleotide sequence identity to the Singapore isolate and 87% identity to the Taiwanese isolate.

## GENOME ANNOUNCEMENT

*Hibiscus rosa-sinensis* is one of the most prevalent ornamental plants grown in private and public gardens. Since 2010, we have identified unfamiliar symptoms on hibiscus plants growing in public gardens across Israel. The disease symptoms include chlorotic spots, yellow rings, and vein banding.

Symptomatic leafs were collected and used as a source material for viral purification as described previously (1). The presence of the viral particles was confirmed by transmission electron microscopy (TEM) (Tecnai G^2^; FEI-Philips, Eindhoven, The Netherlands). Purified virus preparations served as a source material for virion RNA extractions (2). RNA was extracted from the virions and served as a template for a reverse transcription (RT) reaction, first with random hexamer and oligo(dT_17_) primers and later with the sequence-specific reverse complement primers R-HC-424 (5′-TGTCAACCAACCTCCTTTCC-3′) and R-HC-1785 (5′-AAACACCGGCTTCATTTGAC-3′). The obtained cDNA was used to produce double-stranded cDNA (ds-cDNA) fragments that were cloned into pUC19 (which was predigested by SmaI and dephosphorylated) (Fermentas), and both strands of each recombinant plasmid were sequenced (Hylabs, Rehovot, Israel). The obtained nucleotide sequences were subjected to a BLASTn search against the GenBank database and revealed high sequence identity to hibiscus chlorotic ringspot virus (HCRSV), a member of the *Carmovirus* genus with a positive single-strand RNA that putatively encodes seven proteins (3). HCRSV was first identified in 1976 in the United States in *H. rosa-sinensis* plants (4). The complete genome of the Israeli isolate, HCRSV-IL, comprises 3,908 nucleotides and was assembled by sequencing 13 overlapping clones, which covers the entire genome. The HCRSV-IL genome sequence was submitted to GenBank (accession no. KC876666). A BLASTn analysis of the nucleotide sequence of HCRSV-IL with sequences in the GenBank database revealed 93% nucleotide sequence identity to an HCRSV genome isolated in Singapore (accession no. X86448) and 87% sequence identity to the Taiwanese isolate (HCRSV-TW; accession no. DQ392986). Furthermore, HCRSV purified particles were used for mechanical inoculation of *Chenopodium quinoa* and *Hibiscus trionum* plants, using carborundum dust and phosphate buffer (pH 7.0). Symptoms of HCRSV infection appeared 2 weeks after inoculation, producing typical chlorotic spots on *C. quinoa*, while the inoculated *H. trionum* showed chlorotic vein banding. The infections were confirmed by RT-PCR using the HCRSV-specific primers F-HC-3247 (5′-GGCCAATCTGAAGGGGAT-3′) and R-HC-3908 (5′-GGGCTGCCTCACAACTATG-3′). The 647-bp amplicons we obtained were cloned and sequenced to validate the authenticity of the isolate's identification as HCRSV-IL.

### Nucleotide sequence accession number.

The genome sequence of the Israeli isolate of HCRSV has been deposited at GenBank under the accession no. KC876666.
